# *Bifidobacterium*, *Spirulina*, and *Yeast* extracts in low-energy diets for rabbits: effects on performance, hematology, lipid metabolism, hepatorenal function, immunity and hormones

**DOI:** 10.3389/fvets.2025.1615203

**Published:** 2025-07-22

**Authors:** Mohamed S. Shaheen, Ahmed A. Allam, Usama M. Abdel Monem, Sabry M. Bassiony, Bakry A. Khalil, Ayman S. Salah, Abdullah S. Alawam, Hassan A. Rudayni, Ahmed A. Elolimy, Kasim Sakran Abass

**Affiliations:** ^1^Department of Animal Production, Faculty of Agriculture, Zagazig University, Zagazig, Egypt; ^2^Department of Biology, College of Science, Imam Mohammad Ibn Saud Islamic University (IMSIU), Riyadh, Saudi Arabia; ^3^Department of Animal Nutrition and Clinical Nutrition, Faculty of Veterinary Medicine, New Valley University, El-Kharga, Egypt; ^4^Department of Integrative Agriculture, College of Agriculture and Veterinary Medicine, United Arab Emirates University, Al Ain, Abu Dhabi, United Arab Emirates; ^5^Department of Physiology, Biochemistry, and Pharmacology, College of Veterinary Medicine, University of Kirkuk, Kirkuk, Iraq

**Keywords:** feed additives, low energy diet, production, blood metabolites, *Bifidobacterium*, *Yeast* extract, *Spirulina*, rabbit

## Abstract

**Introduction:**

This research assessed the influence of probiotics in low-energy diets on the performance and health status of rabbits during the growing phase. The growth parameters, carcass metrics, serum immunological state, lipid profile, and hepatic and renal functioning of rabbits have been analysed.

**Methods:**

One hundred male New Zealand White rabbits, aged 5 weeks, were split into five groups at random. The rabbits within each group were allocated into 10 replicates, comprising 2 rabbits each. The initial group (T1) received a standard energy diet (10.85 MJ/kg), the (T2) group was provided with a low energy diet (10.25 MJ/kg), whereas the T3, T4, and T5 groups were administered a low energy diet mixed with Bifidobacterium (1 ml/kg diet), Spirulina extract (2 ml/kg diet), and yeast extract (2 ml/kg diet), respectively.

**Results and Discussion:**

The results showed a significant increase in growth performance with the treatment of biological feed additives, and the group treated with spirulina extract increased final body weight and weight gain (5-13 wk), while the group treated with bifidobacterium improved feed intake and feed conversion ratio (5–13 wk). Carcass traits were not significantly affected by probiotic supplementation (*P* > 0.05). Moreover, haematological parameters showed no significant changes (*P* > 0.05) with probiotic supplementation, except for red blood cells (RBCs), white blood cells (WBCs), platelets (PLT), and basophils (BAS), which showed a significant variation (*P* < 0.05). Liver and kidney function tests showed a significant increase (*P* < 0.05) with probiotic treatments. Furthermore, thyroid hormones such as T3 and T4 were significantly enhanced by supplementation of probiotics (*P* < 0.0001). Immunoglobulins (IgA and IgG) were significantly enhanced by supplementation of probiotics when compared to low-energy diet group. Conclusively, probiotics in low-energy diet significantly enhanced rabbit growth, and serum immunity while improving lipid profiles and supporting liver and kidney functions. This supplementation strategy promoted both performance and overall health during the fattening period.

## Introduction

Researchers have been seeking to identify harmless and effective feed additives to boost feed efficiency, production, and economic viability without compromising animal health to address the challenges posed by feed shortages that impede animal output. Probiotics are a medication that may lessen the risk of contamination in birds (1). A variety of probiotics are employed in avian nutrition, like *Lactobacillus and Bifidobacteria* (2, 3), *Lactobacillus* strains (4, 5), and *Saccharomyces cerevisiae* (6). Probiotics have demonstrated the ability to enhance feed conversion ratios and promote weight gain (7, 8), decrease mortality rates (9), mitigate disease infections (10), and stimulate the immune system (11).

*Spirulina* is a widely utilized natural feed additive that functions as a probiotic and comprises bioactive elements. It is safe and cost-effective as a nutritional supplement for people as well as livestock. The SP is utilized as a protein source for human and animal consumption, comprising 60–70% crude protein, all necessary amino acids, and highly digestible minerals (12). It also comprises approximately 1.3–15% important fatty acids (FAs), including myristic, palmitic, oleic, and gamma-linolenic acids, which offer numerous health benefits. Additionally, it serves as a substantial supply of pro-vitamin A and antioxidant qualities (redox status). Recent studies have demonstrated that including microalgae in the diets of rabbits (13), chicks (14, 15), quails (16, 17), various fish species (18, 19), and ruminants (20, 21) enhance feed intake, nutritional digestibility, and overall productive performance.

Yeast, such as *Saccharomyces cervisiae*, has served as an animal feed additive for years. The supplementation of yeast to livestock diets has been revealed to enhance the feed nutritional value and the animal’s productivity (22). Additionally, yeast and mannan oligosaccharides, as well as fructo-oligosaccharides sourced from the cell wall of yeast, have revealed the capacity to suppress enteric pathogens and alter immune responses (23, 24). It was hypothesized that adding biological feed additives to the low-energy diet of fattening rabbits could improve their productive performance and physiological and health status. The current work targeted growing rabbits to study the influence of biological feed additives in the low-energy diet on numerous parameters, including productive parameters, lipid profile, renal function, hepatic function, immunoglobulin levels, and antioxidant status.

## Materials and methods

### Tested probiotics

*Saccharomyces cervisiae* extract: An extract of *Saccharomyces cervisiae* was prepared utilizing a method delineated by Abdel-Rahim et al. (25), which facilitated the efficient growth and proliferation of yeast cells (dry yeast at 100 g/L) under optimal aerobic and nutritional conditions conducive to the synthesis of novel valuable substances such as amino acids, carbohydrates, sugars, proteins, fatty acids, hormones, etc. Subsequently, these substances were liberated from the yeast cells in readily accessible forms through 2 cycles the 1st is freezing followed by thawing to disrupt the yeast cells and release their contents. *Spirulina platensis* extract: The spirulina isolate was obtained from Soda Lake in Wadi el Natrun, Behera governorate, Egypt. *S. platensis* extract was prepared according to Alghamdi et al. (26). *Bifidobacterium: Bifidobacterium* (1 × 10^7^ CFU) was obtained from Agricultural Microbiology, Agriculture Faculty, Zagazig University, Egypt.

### Experimental design, animals and diets

Five different experimental treatments were used in a completely randomized design involving 100 Japanese quail male New Zealand White rabbits, aged 5 weeks. Each treatment comprised ten replications, with each replicate consisting of two rabbits. The experimental design is shown in Table 1.

**Table 1:** Experimental design.

Treatment group	No. of animals	Dietary treatments
T1	20	Standard energy diet-control (10.85 MJ/kg)
T2	20	Low energy diet (10.25 MJ/kg)
T3	20	Low energy diet plus Bifidobacterium (1 ml/kg diet)
T4	20	Low energy diet plus *Spirulina* extract (2 ml/kg diet)
T5	20	Low energy diet plus *Yeast* extract (2 ml/kg diet)

The doses of biological feed additives have been selected according to Mancini and Paci (27). Energy intake on the low-energy diet was reduced by 5.50% compared to the standard energy diet (control group). The cage was supplied with an auto nipple waterer and manual galvanized metal feeders, and its dimensions were 35 × 40 × 60 cm to ensure a constant supply of fresh and clean water and feed. Rabbits were reared under the same management, environmental, and hygienic conditions. Before the trial began, the rabbits were allotted 1 week for acclimatization. The basal feeds were fully pelleted and prepared to meet the necessary nutrient requirements for fattening rabbits, as per NRC (28) (Table 2). The chemical analysis and formulation of the basal feeds administered to rabbits were detailed in the ethics statement, which complies with the rules established by the Ethics Committee of the Egyptian Research for the Utilisation and Welfare of Laboratory Animals at Zagazig University (ZU-IACUC/2/F/313/2023).

**Table 2:** Formulation and chemical analysis of the basal diet fed to rabbits.

Item	Normal energy	Low energy
Ingredient		
Soybean meal	19.00	15.50
Wheat bran	14.50	28.00
Alfalfa hay	20.50	29.00
Peanut hulls	7.500	2.00
Yellow corn	16.00	21.00
Cane molasses	3.00	3.00
Di-calcium phosphate	2.50	2.50
NaCl	0.50	0.50
Premix^1^	0.40	0.40
DL-methionine	0.10	0.10
Total	100	100
Calculated values^2^, as fed		
Digestible energy, MJ/kg	10.85	10.25
Crude protein (N × 6.25)	16.10	16.02
Calcium	0.90	0.88
Phosphorus	0.56	0.54
Crude Fiber	12.01	11.88

^1^Premix provided the following vitamins and minerals per kilogram of diet: vitamin A, 8000 IU; vitamin D_3_, 600 IU; vitamin E, 34 mg; vitamin K_3_, 1.32 mg; vitamin B_1_, 1.32; vitamin B_2_, 4.0 mg; vitamin B_6_, 1.32 mg; vitamin B_12_, 0.01 mg; pantothenic acid, 13.32 mg; biotin, 0.13 mg; folic acid, 3.32 mg; choline chloride, 800 mg; manganese 32 mg; zinc 60 mg; iron 120 mg; copper 16 mg; iodine 2 mg; selenium 0.4 mg; and cobalt 0.4 mg.

^2^Calculated according to tables of ingredients (59).

### Growth performance measurements

The research was extended for 8 weeks from 5 to 13 weeks. The rabbits were weighed separately at 5, 7, 9, 11, and 13 weeks of age (29). At the conclusion of each cycle, the feed intake (FI) of the fattening rabbits was recorded. Consequently, body weight gains (BWG), feed intake (FI), and feed conversion ratio (FCR) were computed (30).

### Carcass traits

At 13 weeks of age, 5 rabbits from each trial group were randomly taken, weighed, and slaughtered to evaluate the carcass characteristics. The carcass, liver, kidney, heart, lungs, spleen, and abdominal fat weights were documented and expressed as a % of the pre-slaughter weight. The dressing percentage and giblets were measured.

### Blood sampling and analysis

At 13 weeks of age, 5 rabbits from each group were used for blood sampling. Two blood samples per animal were collected from rabbits during slaughter. The initial sample was collected in a sterile tube containing an anticoagulant (heparin) for the assessment of hematological parameters in whole blood, as per Brooks et al. (31), utilizing an automated hematology analyzer (Hospitex Hema Screen 18, Sesto Fiorentino, Italy). The 2nd sample was collected, permitted to coagulate, and subsequently centrifuged for 15 min at 1507 g to get serum samples, which were then kept at −20°C until biochemical analysis. The hematological parameters assessed included hemoglobin (Hb, g/dL), red blood cells (RBCs, 10^6^/mm^3^), white blood cells (WBCs, 10^6^/mm^3^), platelets, packed cell volume (PCV, %), hematocrit (HCT), eosinophils (ESO), lymphocytes (LY), monocytes (MON), mean corpuscular volume (MCV), mean corpuscular hemoglobin (MCH), mean corpuscular hemoglobin concentration (MCHC), and sodium (Na). The serum concentrations of albumin (g/dL), total protein (TP g/dL), globulin (GLB g/dL), uric acid (mg/dL), creatinine (mg/dL), and the concentrations of aspartate aminotransferase (AST), and alanine aminotransferase (ALT) (IU/L) enzymes and triglyceride (TG) were quantified utilizing commercial kits (Diamond Diagnostic Company, Giza, Egypt), in accordance with the manufacturer’s guidelines. The serum globulin was determined by subtracting albumin from total protein (globulin = TP - albumin). Thyroid hormones (T3, T4) and immunoglobulins (IgA, IgG mg/mL) were quantified utilizing commercial ELISA kits in accordance with the makers’ directions.

### Statistical analysis

The one-way ANOVA test was employed to examine the data with the subsequent statistical model: Y_ij_ = G_i_ + *μ* + e_ij_.

In this context, Y_ij_ denotes the observation, G_i_ represents the treatment effect (T_1_:T_5_), μ indicates the general mean, and e_ij_ refers to the experimental error. Mean differences were assessed using Tukey’s test. The level of *p* < 0.05 was established as the limit for statistical significance. Statistical analyses were done utilizing SPSS (32).

## Results

### Performance parameters

The influence of nutritional supplements with probiotics on the growth parameters of growing rabbits is displayed in Table 3. The outcomes displayed a substantial increase (*p* < 0.05) in final live body weight and weight gain with probiotic supplementation in low-energy diets, and the group supplemented with *Spirulina* extract displayed the augmented final body weight and body weight gain (2321.67 and 26.89 g) at 13 week and overall (5–13 weeks). Moreover, the feed utilization of fattening rabbits supplied a low-energy diet and treated with biological feed additives revealed a substantial reduction (*p* < 0.05) in feed intake with the supplementation. The group supplemented with *Bifidobacterium* (1 ml/kg diet) displayed a decreased feed intake (56.22 g); in contrast, the group fed low-energy feeds without any supplementation displayed an increased feed intake (70.31 g) along with a trial period of 5–13 weeks. Furthermore, the feed conversion ratio substantially improved (*p* < 0.05) with biological feed additives administration, and the group supplemented with *Bifidobacterium* (1 ml/kg diet) displayed improved feed conversion ratio (2.11); in contrast, the group fed low-energy diets without supplementation displayed the worst feed conversion ratio (3.11) along with a trial period (5–13) week as shown in Table 4.

**Table 3:** Growth performance of growing rabbits as affected by dietary treatments.

Items	Normal energy	Low energy	Low energy +			SEM	*p*-value
			*Bifidobacterium* (1 ml/kg diet)	*Spirulina* extract (2 ml/kg diet)	*Yeast* extract (2 ml/kg diet)		
Live body weight (g) at							
5 weeks	816.7	814.2	815.0	815.8	817.5	12.98	0.9998
7 weeks	1,244^a^	1,180^b^	1,224^ab^	1,235^a^	1,255^a^	15.34	0.0189
9 weeks	1,594^ab^	1,525^c^	1,573^bc^	1,647^a^	1,592^ab^	18.58	0.0027
11 weeks	1,900^b^	1,819^c^	2,020^a^	2,014^a^	1,942^b^	19.11	<0.0001
Final (13 weeks)	2,172^b^	2,082^c^	2,304^a^	2,321^a^	2,265^a^	19.47	<0.0001
Body weight gain (g/day)							
5–7 weeks	30.53^a^	26.13^b^	29.23^a^	30.00^a^	31.31^a^	0.99	0.0146
7–9 weeks	25.00^b^	24.64^b^	24.94^b^	29.41^a^	24.05^b^	0.87	0.0016
9–11 weeks	21.90^c^	21.01^c^	31.97^a^	26.19^b^	25.00^b^	0.95	<0.0001
11–13 weeks	19.40^c^	18.81^c^	20.24^bc^	21.97^ab^	23.04^a^	0.79	0.0048
Overall (5–13 weeks)	24.21^b^	22.65^c^	26.59^a^	26.89^a^	25.85^a^	0.44	<0.0001

^a–c^Means in a column with different superscripts differ significantly. The level of significance was set at *p* ≤ 0.05. The experimental unit was the cage, *n* = 10 cages per treatment. SEM, standard error of the mean.

**Table 4:** Feed utilization of growing rabbits as affected by dietary treatments.

Items	Normal energy	Low energy	Low energy +			SEM	*p*-value
			*Bifidobacterium* (1 ml/kg diet)	*Spirulina* extract (2 ml/kg diet)	*Yeast* extract (2 ml/kg diet)		
Feed intake (g/day)							
5–7 weeks	73.22^a^	69.83^ab^	52.75^c^	62.59^b^	63.39^b^	2.48	<0.0001
7–9 weeks	68.35^a^	77.97^b^	51.82^c^	67.93^b^	57.63^c^	2.20	<0.0001
9–11 weeks	61.80^bc^	67.48^ab^	69.30^a^	67.41^ab^	58.93^c^	1.84	0.0072
11–13 weeks	63.13^ab^	65.93^a^	50.99^c^	56.38^bc^	58.34^b^	2.23	0.0008
Overall (5–13 weeks)	66.63^b^	70.31^a^	56.22^e^	63.58^c^	59.58^d^	0.78	<0.0001
Feed conversion ratio (g feed/g gain)							
5–7 weeks	2.40^b^	2.68^a^	1.80^d^	2.10^c^	2.02^c^	0.07	<0.0001
7–9 weeks	2.75^b^	3.18^a^	2.09^c^	2.31^c^	2.39^c^	0.10	<0.0001
9–11 weeks	2.86^b^	3.23^a^	2.17^d^	2.59^bc^	2.37^cd^	0.10	<0.0001
11–13 weeks	3.26^a^	3.51^a^	2.53^b^	2.58^b^	2.54^b^	0.09	<0.0001
Overall (5–13 weeks)	2.76^b^	3.11^a^	2.11^d^	2.37^c^	2.31^c^	0.04	<0.0001

^a–d^Means in a column with different superscripts differ significantly. The level of significance was set at *p* ≤ 0.05. The experimental unit was the cage, *n* = 10 cages per treatment. SEM, standard error of the mean.

### Carcass criteria

Table 5 displays the carcass traits of fattening rabbits treated with a low-energy diet and probiotics. The data revealed a nonsignificant variation (*p* > 0.05) in carcass criteria with the administration of biological feed additives, especially in the carcass, liver, kidney, heart, lung, head, giblets, and dressing percentage.

**Table 5:** Carcass traits of growing rabbits as affected by dietary treatments.

Items	Normal energy	Low energy	Low energy +			SEM	*p*-value
			*Bifidobacterium* (1 ml/kg diet)	*Spirulina* extract (2 ml/kg diet)	*Yeast* extract (2 ml/kg diet)		
Pre-slaughter weight	2,170^b^	2,080^c^	2,300^a^	2,315^a^	2,255^a^	18.5	<0.0001
Carcass %	75.96	75.36	76.8	76.42	77.65	0.76	0.3653
Liver %	3.19	3.21	3.44	3.53	4.03	0.26	0.3354
Kidney %	0.83	0.71	0.91	0.71	0.81	0.07	0.3707
Heart %	0.53	0.45	0.49	0.48	0.58	0.06	0.7134
Lungs %	0.58	0.61	0.73	0.53	0.65	0.07	0.4801
Head %	6.09	5.95	6.55	6.30	5.97	0.15	0.1272
Giblets %	4.55	4.37	4.83	4.71	5.41	0.26	0.2561
Dressing %	80.51	79.73	81.63	81.13	83.06	0.78	0.1566

^a–c^Means in a column with different superscripts differ significantly. The level of significance was set at *p* ≤ 0.05. The experimental unit was the cage, *n* = 10 cages per treatment. SEM, standard error of the mean.

### Blood hematology

The hematological parameters of growing rabbits treated with biological feed additives are illustrated in Table 6. The finding presented a non-substantial increase in Hb (*p* = 0.2441) with the administration of probiotics. The group treated with *Spirulina* extract (2 ml/kg diet) displayed the highest results (14.87), followed by *Bifidobacterium* (1 ml/kg diet) (14.05), while the energy-fed group demonstrated the lowest results (13.57). Moreover, the findings displayed a substantial variation (*p* < 0.05) in RBCs, WBCs, PLT, and BAS. The group treated with *Spirulina* extract (2 ml/kg diet) displayed the highest RBCs levels (5.43), while the group treated with *Yeast* extract (2 ml/kg diet) displayed increased WBCs levels (5.98). In contrast, the group treated with a low-energy diet revealed decreased RBCs and WBCs levels (4.08 and 4.45). The other hematological parameters, such as HCT, ESO, LY, MON, MCV, MCH, MCHC and NA, presented a non-significant variation (*p* > 0.05) with supplementation with probiotics.

**Table 6:** Hematology of growing rabbits as affected by dietary treatments.

Items	Normal energy	Low energy	Low energy +			SEM	*p*-value
			*Bifidobacterium* (1 ml/kg diet)	*Spirulina* extract (2 ml/kg diet)	*Yeast* extract (2 ml/kg diet)		
HGB (g /dl)	14.34	13.57	14.05	14.87	13.75	0.40	0.2441
RBCs (10^6^/cmm)	4.84^ab^	4.08^c^	4.74^b^	5.43^a^	5.07^ab^	0.19	0.0071
WBCs (10^3^/cmm)	5.11^ab^	4.45^b^	5.84^a^	5.28^ab^	5.98^a^	0.27	0.0192
PLT (10^3^/μl)	188.6^b^	215.6^b^	332.3^a^	226.3^b^	322.6^a^	28.8	0.0197
HCT (%)	41.01	40.56	40.89	41.64	39.27	0.84	0.5542
BAS (10^3^/mm^3^)	0.24^a^	0.15^b^	0.21^a^	0.21^a^	0.19^ab^	0.01	0.0460
ESO (10^3^/mm^3^)	0.34	0.21	0.34	0.36	0.29	0.03	0.1003
LY (%)	72.43	67.20	65.70	69.70	73.09	2.36	0.2103
MON (10^3^/mm^3^)	3.75	3.37	3.29	3.66	3.70	0.47	0.9373
MCV (μm^3^)	89.5	84.8	86.9	79.9	100.9	8.90	0.6643
MCH (pg)	30.15	28.1	29.59	27.93	32.35	1.52	0.4441
MCHC (%)	35.63	35.74	34.07	35.52	32.63	1.88	0.7719
NA (10^3^/mm^3^)	23.00	22.52	34.00	25.67	22.67	5.10	0.6300

^a,b^Means in a column with different superscripts differ significantly. The level of significance was set at *p* ≤ 0.05. The experimental unit was the cage, *n* = 10 cages per treatment.

SEM, standard error of the mean; Hb, hemoglobin; RBCs, red blood cells; WBCs, white blood cells; PCV, packed cell volume; HCT, hematocrit; ESO, eosinophils; LY, lymphocytes; MON, monocytes; MCV, mean corpuscular volume, MCH, mean corpuscular hemoglobin; MCHC, mean corpuscular hemoglobin concentration; NA, neutrophils.

### Serum biochemistry

Dietary supplementation of growing rabbits with probiotics significantly affected (*p* < 0.05) liver and kidney function tests, as illustrated in Table 7. The results presented a significant increase in TP and GLOB (*p* < 0.0001, *p* < 0.0001) in the group supplemented with *Yeast* extract (2 ml/kg diet) (7.09 and 3.42 g/dL) in comparison to the low energy supplemented group (4.55 and 2.16 g/dL). Furthermore, the *Spirulina* extract (2 ml/kg diet) treated group displayed a substantial increase (*p* < 0.0001, *p* = 0.0095) in ALB and A/G % (3.96 and 1.35 g/dL) in comparison to the low energy supplemented group (2.39, 1.11 g/dL). Additionally, AST and ALT significantly decreased (*p* = 0.0032, *p* = 0.0021) with the supplementation of biological feed additives. *Spirulina* extract (2 ml/kg diet) treated group revealed the AST decreased levels (73.42 IU/L), whereas the *Bifidobacterium* (1 ml/kg diet) treated group revealed the ALT decreased levels (150.12 IU/L). Uric acid serum levels displayed a non-significant decrease (*p* = 0.0864) with treatment and the *Bifidobacterium* (1 ml/kg diet) treated group revealed the decreased levels (1.46 mg/dL). In contrast, creatinine levels displayed a significant (*p* = 0.0025) reduction with treatment, and the *Spirulina* extract (2 ml/kg diet) treated group revealed the reduced levels (1.07 mg/dL) relative to the low energy and normal energy treated groups.

**Table 7:** Liver and kidney functions of growing rabbits as affected by dietary treatments.

Items	Normal energy	Low energy	Low energy +			SEM	*p*-value
			*Bifidobacterium* (1 ml/kg diet)	*Spirulina* extract (2 ml/kg diet)	*Yeast* extract (2 ml/kg diet)		
TP (g/dL)	5.31^a^	4.55^c^	5.10^bc^	6.88^a^	7.09^a^	0.21	<0.0001
ALB (g/dL)	2.95^b^	2.39^c^	2.68^bc^	3.96^a^	3.67^a^	0.13	<0.0001
GLOB (g/dL)	2.36^c^	2.16^c^	2.42^c^	2.93^b^	3.42^a^	0.10	<0.0001
A/G (%)	1.26^ab^	1.11^bc^	1.11^bc^	1.35^a^	1.07^c^	0.04	0.0095
AST (IU/L)	89.48^ab^	95.27^a^	81.61^bc^	73.42^c^	80.11^bc^	2.91	0.0032
ALT (IU/L)	168.6^b^	189.8^a^	150.1^c^	162.1^bc^	155.9^bc^	5.01	0.0021
Uric acid (mg/dL)	1.44	1.66	1.46	1.56	1.60	0.05	0.0864
TG (mg/dL)	56.20^bc^	61.31^b^	32.30^d^	45.48^c^	87.50^a^	4.00	<0.0001
Glucose (mg/dL)	186.5^b^	197.95^a^	173.0^c^	163.4^c^	142.2^d^	3.32	<0.0001
Creatinine (mg/dL)	1.32^ab^	1.42^a^	1.20^bc^	1.07^c^	1.26^b^	0.04	0.0025

^a–d^Means in a column with different superscripts differ significantly. The level of significance was set at *p* ≤ 0.05. The experimental unit was the cage, *n* = 10 cages per treatment.

SEM, standard error of the mean; TP, total protein; ALB, albumin; GLB, globulin; A/G, albumin to globulin ratio; AST, aspartate aminotransferase; ALT, alanine aminotransferase; TG, triglyceride.

### Growth hormones and immunity

Dietary treatment of growing rabbits with probiotics meaningfully increased growth hormone (T3 and T4) levels and improved immunity (IgA and IgG) (*p* < 0.0001), as shown in Table 8. *Yeast* extract (2 ml/kg diet) treated group substantially (*p* < 0.0001, *p* < 0.0001) revealed the boosted T3 and T4 levels (1.95 and 1.38). Furthermore, *the Bifidobacterium* (1 ml/kg diet) treated group expressively (*p* = 0.0075) displayed improved IgA levels (0.87), whereas the *Spirulina* extract (2 ml/kg diet) treated group significantly (*p* < 0.0001) displayed improved IgG levels (0.77) relative to low and normal energy treated groups.

**Table 8:** Hormones and immunity parameters of growing rabbits as affected by dietary treatments.

Items	Normal energy	Low energy	Low energy +			SEM	*p*-value
			*Bifidobacterium* (1 ml/kg diet)	*Spirulina* extract (2 ml/kg diet)	*Yeast* extract (2 ml/kg diet)		
T3 (ng/ml)	0.85^d^	0.79^d^	1.57^b^	1.22^c^	1.95^a^	0.08	<0.0001
T4 (ng/ml)	0.74^d^	0.48^e^	1.19^b^	0.93^c^	1.38^a^	0.05	<0.0001
IgA (mg/dl)	0.59^b^	0.57^b^	0.87^a^	0.74^ab^	0.80^a^	0.05	0.0075
IgG (mg/dl)	0.57^b^	0.33^d^	0.47^c^	0.77^a^	0.64^b^	0.02	<0.0001

^a–d^Means in a column with different superscripts differ significantly. The level of significance was set at *p* ≤ 0.05. The experimental unit was the cage, *n* = 10 cages per treatment.

SEM, standard error of the mean; T3 and T4, thyroid hormones; IgA and IgG, immunoglobulins.

## Discussion

This study examined the positive effects of dietary probiotic supplementation, such as *Bifidobacterium, Spirulina extract*, and *Yeast extract*, in the diets of growing rabbits. It demonstrated that food supplementation with yeast yielded beneficial impacts on growth efficacy and health condition. The results established the former findings of several other researchers (33, 34). Ezema and Eze (35) recommended that an additional concentration of 0.12 g of yeast per kg of feed may promote weight gain in the rabbits. The improved growth parameters in rabbits attributed to yeast-containing feed supplements may be due to improved digestion and absorption of feed ingredients (36). It is hypothesized that certain advantages in the growth efficiency of rabbits may result from the beneficial effects of yeast on gastrointestinal health, namely through the enhancement of villus height. Zhang et al. (37) proposed that these results could elucidate the growth-enhancing impact of yeast cell wall constituents on gastrointestinal tract shape. Priya and Babu (38) proved that the incorporation of *Saccharomyces cervisiae* into diets will alter feed digestion, hence improving growth performance. Soliman et al. (39) noted that rabbits consuming a yeast-treated diet achieved markedly greater market weight, increased weight gain, and showed optimal feed conversion efficiency. Conversely, Foad (40) determined that the supplementation of yeast had no impact on body weight (BW), body weight gain (BWG), and feed conversion ratio (FCR) in rabbits.

There is some agreement between the present results and those published by Attia et al. (33), who noted nonsignificant variations in the carcass characteristics of rabbits attributable to dietary yeast administration or mannan oligosaccharides. Shehata et al. (36) reported that the dressing percentage of growing New Zealand White rabbits improved when supplemented with *Saccharomyces cervisiae*. Our findings contradict those of Khanna et al. (34), who stated that the average weights of rabbit carcasses’ fore and hind parts were considerably advanced in yeast-supplemented groups.

The hematology profile offers important findings on the general health of animals. The administration of diverse biological feed additives did not affect blood health, except hemoglobin (Hb), red blood cell (RBC), and white blood cell (WBC) count. Rabbits consuming diets with biological feed additives such as *Bifidobacterium, Spirulina* extract, or *Yeast* extract exhibited elevated hemoglobin levels relative to those on a low-energy diet, suggesting that these substances help mitigate anaemia caused by a low-energy diet in rabbits. Özsoy and Yalçin (41) and Belhassen et al. (42) found that adding active yeast to the feed does not impact the blood parameters of growing rabbits. Attia et al. (33) found similar findings and concluded that dietary supplementation of zinc-bacitracin and mannan oligosaccharides did not substantially modify the hematological characteristics of growing rabbits.

The current finding displayed a significant enhancement (*p* < 0.05) in hepatic and renal function in growing rabbits as an increase in total protein, albumin, and globulin and a decrease in ALT, AST, uric acid, and creatinine with biological feed additive treatments, either *Bifidobacterium* or *Spirulina* extract, or *Yeast* extract. These findings align with the outcome of (43), who reported that the elevation in *Spirulina* concentrations in the T3 and T4 groups in rabbits culminated in considerable (*p* < 0.05) higher serum albumin and substantially minor plasma globulin, urea, creatinine, and ALT concentrations than those in the 2nd and 1st groups. In addition, SP supplementation led to increased levels of TP and ALB, ascribed to its elevated levels of protein, complete amino acids, vitamins, minerals, lipids, and other components (44). However, the decrease in serum GLB level with increasing SP supplementation may be due to the growth inhibition of harmful intestinal bacteria, which reduces inflammatory secretions and globulin synthesis in the liver and other tissues (45). Moreover, the favorable effects of SP addition on serum creatinine and urea levels are related to its functional role as a good influence on feed utilization and kidney activities. Similarly, Ribeiro et al. (46) observed comparable results with rabbits fed SP alone or with yeast diets. The results of AST and ALT are supported by previous research that revealed that SP might work as a preventive agent against liver dysfunctions (47) possibly due to its high contents of different vital nutrients that have numerous positive effects on health. In contrast, some studies found no significant variations in ALT and AST with the addition of zinc or selenium-enriched *Spirulina* (48) to the growing rabbit diets. However, Hassanein et al. (49) stated that *Spirulina* and *Chlorella vulgaris* supplementation in rabbit diets did not affect blood parameters. Additionally, *Spirulina* can activate the immune system due to its rich content of vital organic components, like all amino acids, omega-3 PUFAs, and essential minerals and vitamins for regulating body functions (50, 51). It contains natural active substances that have the potential to function as potent antioxidants, anti-inflammatories, and antiviral agents (52).

The treatment significantly influenced thyroid function, as evidenced by T3 and T4 concentrations in the rabbit’s blood serum. Supplemented rabbits exhibited elevated levels of T3 and T4 when administered Bifidobacterium, *Spirulina* extract, or *Yeast* extract. These findings suggest that the treatments substantially impacted the metabolic hormones of the thyroid gland, indicating typical thyroid action in the supplemented groups. This aligns with the findings of Ansorge et al. (53), who proposed that Propolis exerts an anabolic effect akin to the increase in T3 and T4 observed in groups receiving biological feed additives. Moreover, the present finding demonstrated a substantial enhancement (*p* < 0.05) in immunity parameters (IgA and IgG) in growing rabbits with biological feed additive treatments, either *Bifidobacterium* or *Spirulina* extract or *Yeast* extract. These outcomes align with (54), who described that the administration of SP simulated cytokines, antibodies, and the mobilization of T and B cells. In addition, supplementing SP has been shown to improve most of the immunological parameters of stressed doe rabbits more than any other supplements, either vitamin E or their combination with SP or the control ones El-Ratel et al. (13). However, Mahmoud et al. (55) noted that substituting soybean meal with SP at 20, 40, and 60% in growing rabbit diets exhibited no substantial impact on the immunological parameters. Furthermore, nourishing doe rabbits SP diets improved their productive and reproductive performance and economic efficiency. Our results coordinate with those of Hassanein et al. (49) and (43). A factual demonstration that, under the impact of dietary probiotic supplementation, the healthier gut environment of animals (with its better-balanced microbiota and strengthened mucosa) can function more efficiently was provided by Park et al. (56), who found feed digestion to be enhanced in animals fed probiotic-supplemented diets for 35 days. Besides the exertion of a positive effect on gut health, dietary supplementation of probiotic has been reported to enhance animals’ general health (57, 58).

## Conclusion

The study demonstrates that biological feed additives in low-energy diets effectively boost the well-being and growth performance of rabbits throughout the fattening stage. The significant improvement in final body weight, feed intake, liver and kidney functions, and immunological markers highlights the potential of these additives to optimize rabbit production. Overall, the findings support the use of biological feed additives as a beneficial strategy for improving rabbit welfare and productivity in low-energy feeding systems.

## Data Availability

The original contributions presented in the study are included in the article/supplementary material, further inquiries can be directed to the corresponding author.
